# Ultraviolet Plasmonic Aluminium Nanoparticles for Highly Efficient Light Incoupling on Silicon Solar Cells

**DOI:** 10.3390/nano6060095

**Published:** 2016-05-24

**Authors:** Yinan Zhang, Boyuan Cai, Baohua Jia

**Affiliations:** 1 Centre for Micro-Photonics, Faculty of Science, Engineering and Technology, Swinburne University of Technology, Hawthorn, Victoria 3122, Australia; 2 Institute of Photonics Technology, Jinan University, Guangzhou 510632, China; caiboyuan@jnu.edu.cn

**Keywords:** ultraviolet plasmonics, aluminium nanoparticles, light incoupling, light trapping, solar cells

## Abstract

Plasmonic metal nanoparticles supporting localized surface plasmon resonances have attracted a great deal of interest in boosting the light absorption in solar cells. Among the various plasmonic materials, the aluminium nanoparticles recently have become a rising star due to their unique ultraviolet plasmonic resonances, low cost, earth-abundance and high compatibility with the complementary metal-oxide semiconductor (CMOS) manufacturing process. Here, we report some key factors that determine the light incoupling of aluminium nanoparticles located on the front side of silicon solar cells. We first numerically study the scattering and absorption properties of the aluminium nanoparticles and the influence of the nanoparticle shape, size, surface coverage and the spacing layer on the light incoupling using the finite difference time domain method. Then, we experimentally integrate 100-nm aluminium nanoparticles on the front side of silicon solar cells with varying silicon nitride thicknesses. This study provides the fundamental insights for designing aluminium nanoparticle-based light trapping on solar cells.

## 1. Introduction

Light management is of critical importance for constructing high efficiency solar cells. Conventionally, micro-scale textured surfaces, such as pyramid structure, have been widely employed to improve the light absorption in Si solar cells. Textured surfaces not only reduce the surface reflection by increasing the chance of the reflected light bounced back to the Si layer but also enhance the light trapping through the redirection of the incident light. Although they have been well demonstrated and applied on the commercial solar cells, they induce a few drawbacks as well. Firstly, textured surfaces increase the surface area of the solar cells, leading to an increase of the surface recombination and thus the reduction of the solar cell performance. Secondly, they increase the total volume of the depletion junction region. Thirdly, they are not suitable for the light management in ultra-thin solar cells, which have been identified as one of the most effective strategies for the cost reduction of the solar cells.

Recently, nano-scale light management strategies, particularly the plasmonic nanoparticles (NPs) and nanostructures have emerged and attracted a great deal of interest [1,2,3]. Metal NPs, supporting localized surface plasmons, exhibit both far-field scattering and near-field light concentration due to the collective oscillation of the free electrons in the metal [4,5]. Both effects can contribute to the light absorption enhancement of solar cells. By tuning the geometry parameters of the NPs, the strength of light scattering/concentration and the resonance wavelength can be adjusted accordingly. Nanoparticles with a size smaller than 20 nm show strong near-field light concentration in their immediate vicinity, which can potentially enhance the light absorption in this region. However, so far, all of the thicknesses of the active layers of the solar cells are a few times larger than the near-field light concentration regions of the NPs. Therefore, the near-field light concentration is hardly able to increase the absorption of the solar cells. On the other hand, the scattering of the particles has been the main mechanism used for light absorption enhancement. Nanoparticles larger than 100 nm show strong scattering strength. Once they are placed on the surface of the solar cells, light will be preferentially scattered into the high-index substrates. This increases the photon absorption in solar cells in at least two ways. Firstly, the preferential scattering reduces the light reflection due to the optical impedance matching, leading to an enhanced light incoupling into the solar cell. Secondly, light is redistributed inside the solar cells as a result of the scattering, leading to an increased light path length, which is particularly useful for the weakly absorbed near-bandgap energy.

At the early stage of the plasmonic solar cell research, the noble metal Ag and Au NPs were widely studied and demonstrated to be effective in trapping the near bandgap energy due to higher radiative efficiency [6,7,8,9,10,11,12,13,14,15,16,17,18,19,20,21,22,23,24,25]. However, a transmission reduction is always found at the short wavelength of the usable solar spectrum (300–1200 nm) due to the Fano effect, *i.e.*, the destructive interference between the incident light and the scattered modes at the wavelengths below the surface plasmon resonances (SPRs) [26]. Furthermore, the noble metal Ag and Au are expensive, limiting their real-life applications. Al NPs, with their SPRs spanning from visible to ultraviolet region, can potentially blueshift this reduction away from the usable solar spectrum, thereby leading to a broadband light incoupling [27,28,29,30]. Together with the low cost, earth-abundance, and their high compatibility with the complementary metal-oxide semiconductor (CMOS) manufacturing process, Al NPs hold great promises for the next-generation plasmonic solar cells. It should be mentioned here that embedding the NPs in a thin dielectric layer can also avoid this detrimental Fano effect, providing another way to mitigate this light incoupling reduction at the short wavelengths [31].

**Figure 1:**
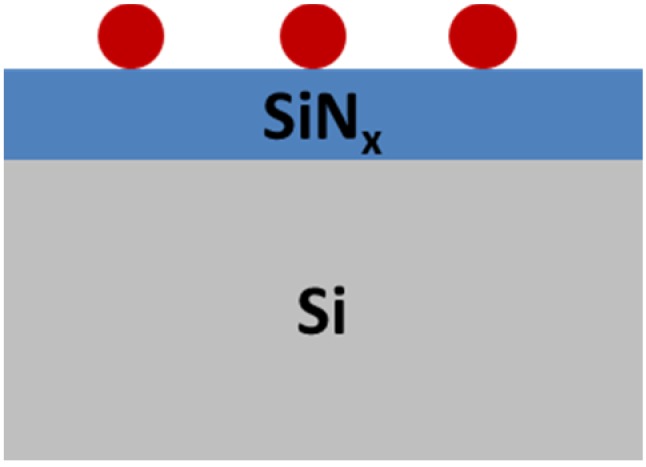
Schematic diagram of the investigated solar cell structure, consisting of the Al nanoparticles (NPs), the SiN*_x_* spacing layer, and the Si layer with the red spheres representing the metal NPs.

So far, Al NPs have been applied to many types of solar cells, such as Si wafer solar cells [32], thin-film GaAs solar cells [33], thin-film Si solar cells [34,35], GaInP/GaInAs/Ge triple junction solar cells [36], dye-sensitized solar cells [37], and organic solar cells [38], making them a rising star in this field. However, there is no systemic study on the critical parameters of the Al NP array, which is of importance in designing and optimizing their light incoupling. Here, in this paper, we report some key factors that determine the light incoupling by the Al NPs located on the front side of Si solar cells. Figure 1 schematically shows the structure of the solar cells, consisting of the Al NPs, the SiN_x_ spacing layer, and the Si layer. We first numerically study the scattering and absorption properties of the Al NPs and the influence of the NP shape, size, surface coverage, and the spacing layer on the light incoupling using the finite difference time domain (FDTD) method. Followed this, we experimentally integrate 100-nm Al NPs on the front side of Si solar cells with varying SiN*_x_* spacing layer thicknesses.

## 2. Results and Discussion

### 2.1. Broadband Light Incoupling by Al NPs

**Figure 2:**
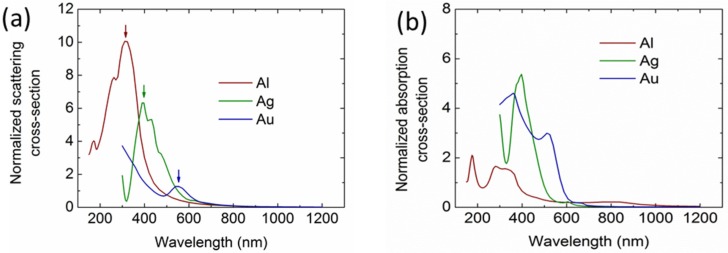
Calculated normalized scattering (**a**) and absorption (**b**) cross sections of the Al NPs with a 100-nm diameter on top of a Si layer, in comparison with Ag and Au NPs.

To investigate the light incoupling properties, it is important to understand the scattering and absorption properties of the Al NPs in comparison to other widely used metal NPs, including Ag and Au. Figure 2a,b illustrate the normalized scattering and absorption cross sections of the three types of metal NPs (100-nm diameter) on top of a Si layer, with the arrows indicating their dipole resonance wavelengths. Clearly, the SPRs of the Ag and Au NPs both lie in the visible ranges at around 400 nm and 550 nm, respectively, whereas the Al NP shows an ultraviolet resonance at around 300 nm. The absorption cross sections of the NPs (Figure 2b) demonstrate an associated absorption loss, particularly at the resonance region. It should be noted that the slightly large absorption at around 800 nm for Al NPs is introduced by the interband transition.

**Figure 3:**
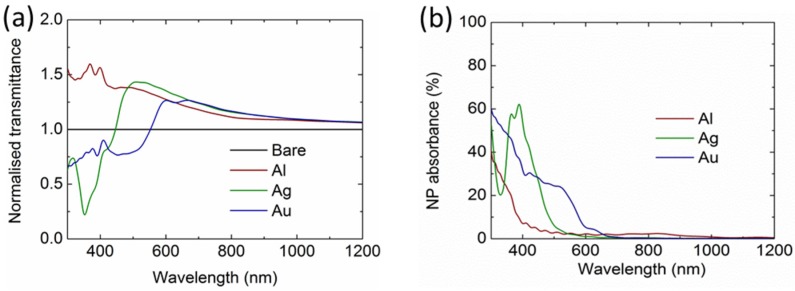
(**a**) Calculated normalized light transmittance of Si solar cells integrated with an array of Al NPs (100-nm diameter and 10% surface coverage), compared with the Si solar cells integrated with Ag and Au NPs. (**b**) Calculated absorption losses in the NP arrays.

To demonstrate the superiority of the Al NPs in light incoupling, we simulated the light transmittance into the solar cells integrated with an ordered periodic NP array with a 100-nm diameter and 10% surface coverage (280-nm pitch) for all the three materials: Al, Ag, and Au. In an ordered array, far-field diffraction occurs, leading to a redistribution of the scattered light and change of the light incoupling spectra, whereas the total scattering and incoupling of the NPs in a random array is generally the sum of that for each NP, provided that the surface coverage is low enough [23]. Figure 3a shows the normalized transmittance of the solar cell integrated with NPs. As can be seen, the light transmittance into Si has been increased at wavelengths above the SPRs for Ag and Au NPs, with the largest enhancement up to 43% at 510 nm and 26% at 600 nm, respectively. However, at short wavelengths, the light transmittance has been largely reduced. For Al NPs, the light transmittance has been increased among the entire wavelengths from 300 to 1200 nm without any reduction, demonstrating a broadband light incoupling enhancement. Figure 3b shows that Al NPs also perform better in terms of loss control at the wavelengths below 600 nm, except at around 800 nm with a minor absorption.

### 2.2. Shape Study of the Al NPs

**Figure 4:**
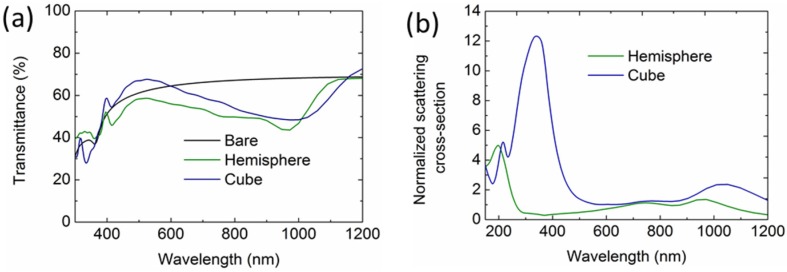
(**a**) Calculated transmittance of the Si solar cells with an array of hemispherical and cubic Al NPs (100-nm diameter/width and 10% surface coverage), referenced to that of a bare Si layer. (**b**) Calculated normalized scattering cross sections of the corresponding Al NPs on top of the Si layer.

We have demonstrated that the Al NPs show broadband light incoupling, compared with Ag and Au NPs. In this section, we study the influence of the shape of the Al NPs on the light incoupling in the Si wafer solar cells. We simulated the light transmittance of a Si wafer with an ordered hemispherical (100-nm diameter) and cubic (100-nm width) Al NP array with 10% surface coverage, with the results shown in Figure 4. It is remarkable and interesting that the transmittance of the Si wafer with the hemispherical and cubic Al NPs reduces at the longer wavelengths (Figure 4a), compared with that of the bare Si wafer, which is attributed to the redshifts of the Fano resonance for the dipole scattering modes. To understand this, we calculated the scattering cross sections of the hemispherical and cubic Al NP on top of a Si layer, as shown in Figure 4b. Clearly, the dipole resonance shifts to around 1050 nm and 960 nm for the hemispherical and cubic NPs due to the large contact area between the NPs and the high index Si substrate. Therefore, the light incoupling reduction shifts to the long wavelengths. This is distinct from the broadband light incoupling of the 100-nm spherical Al NPs with only a point contact with the Si layers.

### 2.3. Size and Surface Coverage Effect

**Figure 5:**
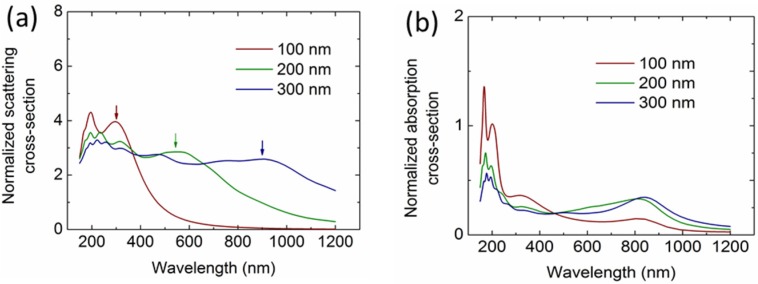
Calculated normalized scattering (**a**) and absorption (**b**) cross sections of the Al NPs in the air with 100-nm, 200-nm and 300-nm diameters, respectively.

Since spherical Al NPs have been demonstrated to be more effective in light incoupling than the other shapes, this section studies the two key parameters in optimizing the spherical Al NP array, *i.e.*, the diameter and the surface coverage. Before investigating their influence on the light incoupling, we calculated the scattering and absorption of the Al NPs with different sizes. Figure 5a,b give the normalized scattering and absorption cross sections of the spherical Al NPs with varying diameters. As can be seen, the dipole resonance scattering (indicated with arrows) redshifts and broadens when the particle size increases. At the same time, higher order resonances are excited at the short wavelengths and also redshift. For the absorption, it is found that the interband absorption peaks at around 200 nm do not shift, but the strength reduces when the particle size increases. However, the absorption at around 800 nm increases when the particle size increases, which is distinct from the phenomena observed in the widely used Ag and Au NPs. This is possibly due to the fact that the dipole resonances shift to this region, leading to a resonance-related near-field light confinement, which overlays onto the interband absorption of the Al NPs.

**Figure 6:**
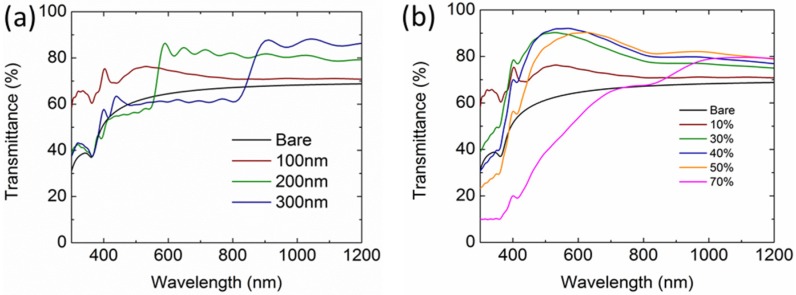
Calculated light transmittance into the Si wafer under the configurations of (**a**) varying diameter at 10% surface coverage and (**b**) increasing surface coverage from 10% to 70% for a 100-nm particle size.

Figure 6a,b present the light transmittance of the Si solar cells integrated with an array of Al NPs under the configurations of varying diameter at 10% surface coverage and increasing surface coverage from 10% to 70% for a 100-nm particle size. Compared with 100-nm Al NPs, 200-nm and 300-nm Al NPs induce a transmission reduction region at short wavelengths due to the redshifts of the dipole scattering-related Fano resonance (Figure 5a). However, the transmission becomes larger at the longer wavelengths owing to the increased scattering cross section when the particle size increases. With the surface coverage increasing, the transmission increases at first and then decreases, reaching a maximum value (>90%) at 600 nm for 40% surface coverage. When the surface coverage increases beyond 40%, the particle interplay becomes severe, leading to redshifts of the dipole scattering modes; therefore, the Fano resonance-related transmission reduction occurs. The optimization of the size and the surface coverage would be a result of the spectra shifts matching the peak intensity of the standard AM1.5G solar photon fluxes. The optimized enhancement could be around 28.7% for the Al NPs under the configuration of a 150-nm diameter and 30% surface coverage. By further tuning the particle height and width, the enhancement can be as high as 30%. This is far higher than that induced by the optimized Ag (27.2%) and Au (14.5%) NPs.

### 2.4. Study on the Thickness of SiN_x_ Spacing Layer

**Figure 7:**
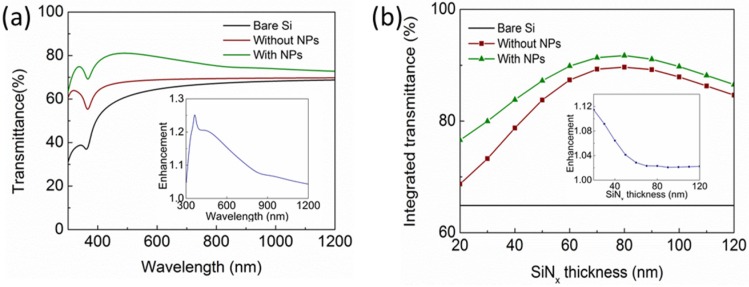
(**a**) Calculated light transmittance spectra of the Si wafer integrated with the Al NP array (100-nm diameter and 10% surface coverage) on top of the 20-nm SiN*_x_*, referenced to that without Al NPs and the bare Si. (**b**) Calculated integrated light transmittance of the Si wafer with the Al NPs as a function of the SiN*_x_* spacing layer thickness. The inset figures show the relative enhancement.

The spacing layer between the Al NPs and the Si layer plays an important role in determining the incoupling efficiency induced by the Al NPs since it affects the distance between the excited dipole moment in the NPs and the underlying substrate. This section gives some study on the influence of the spacing layer thickness on the Al NPs incoupling, using the SiN*_x_* spacing layer as an example. The light transmittance into the Si layer by the Al NP array, using an example configuration of a 100-nm diameter and 10% surface coverage, was calculated with varying thicknesses of the SiN*_x_* layer from 20 nm to 120 nm. Figure 7a shows the transmittance spectra of the Si wafer integrated with the Al NPs on top of 20-nm SiN*_x_*. As shown, the 20-nm SiN*_x_* boosts the transmittance across the entire wavelength band from 300 nm to 1200 nm. It acts as an antireflection layer, making use of the destructive interference between the reflected light from the air/SiN*_x_* and SiN*_x_*/Si interfaces. By adding the Al NPs, the transmittance is further increased, with an enhancement up to 25% at the wavelength around 400 nm compared with that of the solar cells with 20-nm SiN*_x_* solely. Figure 7b gives the integrated transmittance at different thicknesses of SiN*_x_* layer by weighting the transmittance to the AM1.5G solar photon fluxes. Clearly, as the SiN*_x_* thickness increases from 20 nm to 120 nm, the integrated transmittance firstly increases and then reduces, reaching a maximum transmittance at 80-nm SiN*_x_* for both the cells with and without NPs. However, the enhancement (the ratio of the integrated transmittance of the solar cells with NPs to that without NPs) reduces from around 12% to 2%, as shown in the inset of Figure 7b, demonstrating that thicker spacing layer reduces the light incoupling efficiency.

### 2.5. Experimental Demonstration

**Figure 8:**
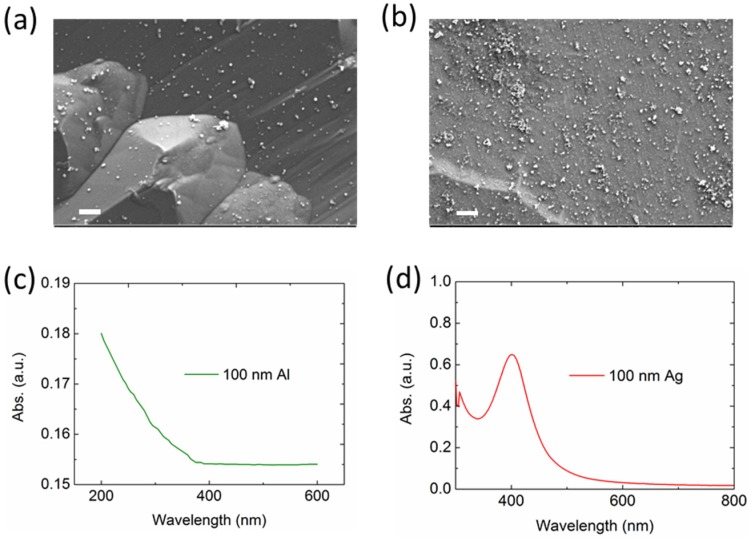
Scanning electron images (SEM) of the fabricated Al (**a**) and Ag (**b**) NPs (Scale bar: 1 µm). The UV-VIS-NIR spectra of the Al (**c**) and Ag (**d**) NPs in deionized water solution.

As a demonstration, we experimentally fabricated the Al and Ag NPs with a 100-nm average size and integrated them on the front side of the bare Si solar cells by the spray-coating method. Before the integration, we measured their morphologies and optical properties. Figure 8a,b present the scanning electron images (SEMs) of the prepared Al and Ag NPs, with their extinction spectra shown in Figure 8c,d, respectively. As shown, the particles are randomly distributed on the surface of the Si and aggregate in some regions, which slightly affects the particle resonances and the light incoupling. The SPRs for Al and Ag NPs lie in below 300 nm and around 400 nm, respectively, which agree well with the above-mentioned simulation.

**Figure 9:**
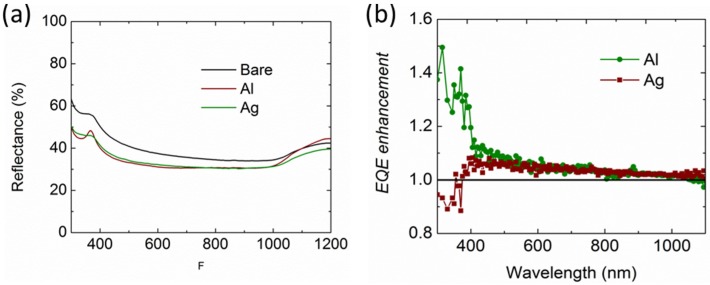
(**a**) Measured reflectance of the Si solar cells integrated with Al and Ag NPs. (**b**) Measured external quantum efficiency (EQE) enhancement of the solar cells integrated with Al and Ag NPs, relative to that without NPs.

**Figure 10:**
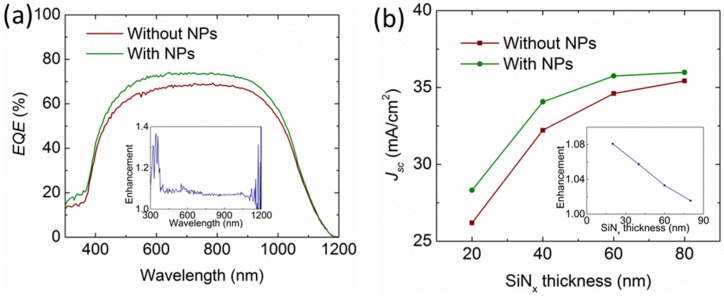
(**a**) Measured EQE of the solar cells with the Al NPs on top of a 20-nm SiN_x_ spacing layer, compared with that without Al NPs. (**b**) The corresponding photocurrent density as a function of the SiN_x_ layer thickness. The inset figures are the relative enhancement.

Figure 9a,b show the reflectance and the normalized external quantum efficiency (EQE) of the solar cells integrated with Al and Ag NPs without the SiN_x_ layer, respectively. It is noted that the reflectance for both solar cells reduces among the entire wavelength region. However, the EQE enhancement for the Al NPs is broadband over the entire spectrum with an enhancement up to 40% at the short wavelengths, whereas that for the Ag NPs is found to reduce at the short wavelengths below 400 nm. This agrees well with the simulated light transmittance shown in Figure 3a. Then, the Al NPs were integrated on the front surface of the solar cells with various SiN*_x_* thicknesses at 20 nm, 40 nm, 60 nm, and 80 nm, respectively. The EQE results and the corresponding photocurrent density are shown in Figure 10a,b, respectively. As can be seen, the EQE is increased among the entire wavelengths from 300 nm to 1200 nm, even with a 20-nm-thick SiN*_x_* layer, agreeing well with the light transmittance shown in Figure 7a. Accordingly, the photocurrent was increased from 26.2 mA/cm^2^ to 28.3 mA/cm^2^, representing an enhancement of 8%. In Figure 10b, the photocurrents of the solar cells integrated with and without Al NPs both increase when the SiN_x_ thickness increases from 20 nm to 80 nm. However, the enhancement induced by the Al NPs shown in the inset figure shows a saturated trend due to a reduced light incoupling. The largest photocurrent achieved is 36 mA/cm^2^ with Al NPs on top of the 80-nm-thick SiN*_x_* layer.

## 3. Materials and Methods

### 3.1. Numerical Simulations

Lumerical FDTD solutions (Lumerical Solutions, Inc., Vancouver, Canada) was employed to perform the optical simulation. For the scattering/absorption cross-section calculation, a 3-D total-field scattered-field (TFSF) source from 300 nm to 1200 nm was used to surround a NP. Two analysis groups, comprising 6 transmission monitors for each group in 3-D, were placed inside and outside the light source box, respectively. The inside and outside groups obtain the absorption power and the scattering power, respectively. The boundary conditions were set as the perfect matcher layers (PMLs) to prevent the scattering light bouncing back to the NPs from the boundaries, leading to an interference effect. For the light incoupling calculation, a spherical NP was placed on the surface of a planar Si slab with and without a SiN*_x_* spacing layer and illuminated under a 300–1200-nm plane wave source weighted against the AM1.5G solar spectrum. PML boundary conditions were used in the incident direction to prevent an interference effect, and periodic boundary conditions (PBCs) were used in the lateral direction to simulate an ordered array of NPs.

### 3.2. NP Fabrication and Characterization

Due to the strong activeness of the Al NPs, which can be easily oxidized, it is challenging to synthesize Al NPs using the conventional chemical reduction method. To overcome this challenge, we developed a novel fabrication process based on the conventional thermal evaporation and annealing process. An ultra-thin Al film, with a few nanometers in thickness, was evaporated on the NaCl powder substrate, followed by a high temperature annealing process. Then, the powder was dissolved in the deionized water, and the Al NPs were separated from the water solution by centrifugation. The particle size can be adjusted by tuning the thickness of the Al film and the annealing condition. In this paper, 10-nm Al film was evaporated and annealed in a nitrogen atmosphere at 400 °C to form the Al NPs with the desired size (around 100 nm in diameter). As a remark, the Al itself is easy to be oxidized, leading to a thin layer of alumina on the surface of the Al NPs, preventing it from being further oxidized. This thin layer of alumina is only a few nanometers, as shown in [29], and would not affect the optical properties of the Al NPs much. For the purpose of comparison, we also synthesized 100-nm Ag NPs by the NaBH_4_ reduction of silver nitrate solution [39]. A 5-mL deionized water solution with 5 mM AgNO_3_ and 5 mM sodium citrate were prepared. Next, the suspension was subjected to the sonication. A 0.6-mL portion of 50 mM freshly prepared NaBH_4_ was injected quickly at room temperature, followed by 30 s of sonication. Then, the solution was centrifuged at 5000 rpm for 10 min, and the supernatant was removed and the precipitate, containing the Ag NPs was redispersed in deionized water. The UV-VIS-IR spectrum of the NPs in the solution was measured by the spectrophotometer (Lambda 1050, PerkinElmer, Waltham, MA, USA).

### 3.3. Solar Cell Fabrication and Characterization

The planar Si solar cells were fabricated by the following processes. 200-μm thick Si wafers with resistivities around 1–3 Ω·cm were used. Firstly, the saw-damaged surface of the wafer with around 20 μm were etched off by dipping the wafers into the HNO_3_/HF solution. After the standard RCA cleaning (as developed at Radio Corporation of America) process, the n^+^ emitters were formed by a phosphorus diffusion process using the POCl_3_ source at the temperature around 850 °C. The phosphosilicate glass introduced by the diffusion process was removed by the HF solution. Then, Al paste was screen printed on the rear surface and fired at a temperature around 800 °C for a few seconds to form the back surface field (BSF) and the back contact. An Al front contact grid was formed by the photolithography. After that, the SiN*_x_* ARC was deposited by the plasmon-enhanced chemical vapour deposition (PECVD) system at 350 °C. The EQE of the solar cells was measured across 300–1200 nm using PV Measurements QEX10 (PV Measurements, Boulder, CO, USA). The photocurrent density was calculated by integrating the EQE to the AM1.5G solar photon fluxes.

## 4. Conclusions

In conclusion, we have demonstrated highly efficient light incoupling by Al NPs and have revealed the key factors that determine the incoupling efficiency, including the shape, size, surface coverage, and the spacing layer through numerical simulation. Furthermore, we experimentally compared the performance of the Si solar cells integrated with and without the Al NPs on top of the SiN_x_ spacing layer at varying thicknesses.
